# Comparative Analysis of Cannabidiol and Risperidone on Behavioral and Neurochemical Outcomes, and Neurodevelopment Markers in a Zebrafish Model of Embryonic Exposure to Sodium Valproate

**DOI:** 10.1002/aur.70151

**Published:** 2025-11-26

**Authors:** Karla C. M. Costa, Tamires A. V. Brigante, Pedro H. C. Lirio, Gabriel G. Fernandes, Franciele F. Scarante, Davi S. Scomparin, Rafael R. Ferreira, Maria A. Vicente, Flavia R. Abe, Francisco S. Guimarães, Jaime E. C. Hallak, Jose A. Crippa, Danielle P. de Oliveira, Alline C. Campos

**Affiliations:** ^1^ Department of Pharmacology Ribeirão Preto Medical School, University of São Paulo Ribeirão Preto SP Brazil; ^2^ Center for Cannabinoid Research, Mental Health Building Ribeirão Preto Medical School, University of São Paulo Ribeirão Preto SP Brazil; ^3^ Department of Clinical Analyses, Toxicology and Food Science School of Pharmaceutical Sciences of Ribeirão Preto, University of São Paulo São Paulo SP Brazil; ^4^ National Institute of Science and Technology for Translational Medicine (INCT TM), CNPQ/FAPESP/CAPES Ribeirão Preto SP Brazil; ^5^ Department of Neuroscience and Behavior Ribeirão Preto Medical School, University of São Paulo São Paulo SP Brazil; ^6^ Laboratory of Psychiatric Neuroimaging (LIM21) Hospital das Clínicas da Faculdade de Medicina da Universidade de Sao Paulo (HCFMUSP) São Paulo SP Brazil

**Keywords:** autism spectrum disorder, cannabidiol, endocannabinoids, lipid peroxidation, risperidone, zebrafish

## Abstract

Autism spectrum disorder (ASD) is a neurodevelopmental condition characterized by social communication deficits, repetitive behaviors, and sensory abnormalities. Sodium valproate (VPA) exposure during embryonic development is a well‐established preclinical model for ASD, leading to increased oxidative stress in the developing brain, including lipid peroxidation, which affects cell proliferation and organization. This study aimed to investigate the potential therapeutic effects of cannabidiol (CBD) and risperidone (RISP) in reversing ASD‐like behaviors and associated neurobiological alterations induced by embryonic VPA exposure in a zebrafish model. Zebrafish embryos were exposed to 125 μM VPA for 2 days post‐fertilization (dpf). At 3–4 dpf, embryos were treated with 0.06 μM CBD or 1 μM RISP. Behavioral assays were conducted to assess hyperlocomotion and aggressive behavior. At 7 dpf, lipid peroxidation levels were measured, and expression of glial fibrillary acidic protein (GFAP) and calcium/calmodulin (CaM) were analyzed to evaluate neurobiological changes. VPA exposure resulted in increased hyperlocomotion and aggression. CBD treatment effectively reversed these behaviors, while RISP showed limited efficacy. Additionally, CBD reduced lipid peroxidation and restored anandamide levels, whereas RISP did not exhibit these effects. CBD also normalized GFAP and CaM expression, indicating restoration of glial function and excitatory/inhibitory balance. CBD demonstrated a better efficacy and safety profile compared to RISP in reversing ASD‐like behaviors and associated neurobiological alterations in the zebrafish model. These findings suggest that CBD may offer a safer and more effective therapeutic alternative for managing ASD‐related symptoms.

## Lay Summary

In our study, we compare the effects of cannabidiol (CBD), a compound from the cannabis plant, and risperidone, an antipsychotic drug, in attenuating autism‐like behaviors in zebrafish. A low dose of CBD improved aggressive behavior caused by early‐life exposure to sodium valproate, a known risk factor for the precipitation of autism spectrum disorders (ASDs). CBD also reduced signs of brain oxidative stress without disrupting important brain chemicals, unlike traditional treatments like risperidone. These results suggest CBD may offer a safer, promising approach to help manage ASD symptoms.

## 1 Introduction

Autism spectrum disorder (ASD) is a complex neurodevelopmental condition marked by a wide range of clinical manifestations and severity. Symptoms include deficits in social–emotional reciprocity, executive function impairments, communication difficulties, and sensory processing challenges (Lord et al. 2018). Recent data report that ASD affects about 1 in 36 children, being 4.3 times more prevalent in males (Maenner et al. 2023). Additionally, ASD is often accompanied by intellectual disability, aggression, hyperactivity, social anxiety, epilepsy, sleep disturbances, and gastrointestinal symptoms (Lord et al. 2018; Pedrazzi et al. 2022).

Besides genetic factors and abnormal brain development during critical periods (LeBlanc and Gillis 2012; Medina et al. 2023), environmental stressors, infections, and teratogenic drugs, including sodium valproate (VPA), are recognized as risk factors (Karimi et al. 2017) for ASD (Christensen et al. 2013). Prenatal VPA exposure is a well‐established model for studying ASD‐like features in rodents (Schneider and Przewłocki 2005; Wagner et al. 2006), due to its effects on oxidative stress balance, histone acetylation, serotonin turnover, and excitatory/inhibitory balance during neurodevelopment (Fereshetyan et al. 2021; Mabunga et al. 2015; Nicolini and Fahnestock 2018).

Despite major advances in the understanding of factors that might increase vulnerability to ASD, the pharmacological treatment remains suboptimal, focused on the relief of specific symptoms (Aishworiya et al. 2022). In this context, antipsychotic drugs such as risperidone (RISP) and aripiprazole are the only FDA‐approved medications for ASD patients, but they exhibit limited efficacy depending on the severity of the ASD as reported across clinical studies (Broadstock et al. 2007; Murray et al. 2015).

Recently, CBD, the main non‐psychotomimetic phytocannabinoid derived from *Cannabis sativa* , has shown promise in improving neuropsychiatric symptoms in ASD patients (Hacohen et al. 2022; Pretzsch et al. 2019; Silva Jr et al. 2024) and cognitive function in mouse models of ASD (Pedrazzi et al. 2025). Additional studies also support the efficacy of CBD‐enriched extracts in enhancing sociability in individuals with ASD (Barchel et al. 2019; Bilge and Ekici 2021; Silva Jr et al. 2024). However, the characterization of the mechanism associated with CBD effects remains poorly understood in the context of ASD.

In this study, we developed a rapid and reproducible platform for ASD drug screening by inducing persistent ASD‐like traits through embryonic exposure to VPA in zebrafish. Indeed, different zebrafish VPA‐ASD study models have been developed through different exposure methods, evaluating changes in the molecular, proteomic, cellular, and behavioral patterns (for an extensive review, please read Camussi et al. 2025). Additionally, we explore possible mechanisms associated with the long‐term behavioral, neurochemical, and neurodevelopmental effects of CBD and RISP during the larval stages.

## 2 Material and Methods

### 2.1 Animals

Wild type zebrafish ( *Danio rerio* ) embryos and larvae were obtained from the facility at the School of Pharmaceutical Science of Ribeirão Preto, University of São Paulo (Ribeirão Preto, Brazil). The adults were kept in a ZebTEC (Tecniplast, Italy) recirculating system using water obtained by a reverse osmosis system at standard conditions (pH 7.5 ± 0.5, temperature 26°C ± 1°C, dissolved oxygen at 95% saturation, and conductivity 750 ± 50 μS/cm, 14 h:10 h light/dark cycle). The experimental protocols were approved by the Ethics Committee on Animal Use at the University of São Paulo (CEUA/FCFRP no. 19.1.243.60.4). Fertilized eggs obtained by placing male and female breeding stock in a 2:1 ratio in a 1 L aquarium, were washed using freshly prepared embryo medium (2 mM CaCl_2_⋅2H_2_O, 0.5 mM MgSO_4_⋅7H_2_O, 0.75 mM NaHCO_3_, and 0.07 mM KCl) and observed under a stereomicroscope (Zoom Microscope SMZ 1500, Nikon), and those in the cleavage stage (4–64 cells) were randomly selected for experiments. During the first 120 h post‐fertilization (hpf), the larvae feed from their yolk sac, eliminating the need for additional feeding during the experiments. At 6 days post‐fertilization (dpf), external feeding was offered (microflocculated larval food; Alcon). Before the experimental assays, larvae were fasted overnight.

### 2.2 Drugs

Sodium valproate (VPA; Prati‐Donaduzzi, Brazil), RISP (Prati‐Donaduzzi, Brazil) freshly prepared in stock solutions of VPA (12.5 mM), and RISP (0.03 mM) using ultrapure water were used. The stock solution of CBD (99.9% pure CBD, BSPG Pharm, Brentwood, UK) (9.54 mM) was prepared in DMSO. To avoid DMSO‐induced toxicity, all experiments were performed with DMSO diluted at 0.01% (v/v) (ISO 7346‐3, 1996) (Brigante et al. 2018).

### 2.3 Experimental Procedures

#### 2.3.1 Embryonic Exposure to VPA

Fish embryo toxicity (FET) test was conducted (OECD 2025) to evaluate the impacts of the VPA during development. For that, eggs were randomly and individually placed in 24‐well plates, containing 2.0 mL of tank water + each VPA concentration (5, 25, 125, 625, and 1250 μM). Exposure of VPA occurred for 48 h; lethality (egg coagulation, non‐detached tail, absence of somites, and no heartbeat), sublethality (eye development, spontaneous movements, blood circulation, pigmentation, and swim bladder inflation), and teratogenicity (malformed head, otoliths, tail, or heart; scoliosis; yolk sac deformity; and altered growth) were evaluated daily (Nagel 2002) using a stereomicroscope (Stemi 508, Carl Zeiss, Germany) equipped with a camera (AxioCam ICc 5, Zeiss, Germany).

To assess effects on spontaneous embryonic movement, the tail coiling activity test was accomplished as previously reported (de Oliveira et al. 2021). Tests were conducted in triplicate. At 24 hpf, non‐hatched embryos from each group exposed to VPA (5, 25, 125, 625, and 1250 μM) were grouped on an excavated slide and placed under a stereomicroscope (Stemi 508, Zeiss, 0.63). After a 5‐min acclimation period, tail coiling behavior was recorded for 3 min using Zen 2.3 Image software (Zeiss) and videos recorded were processed using DanioScope software (Noldus), which computed the burst activity frequency, defined as the percentage of time the embryo exhibited spontaneous movement (*n* = 20 per group, three independent replicates).

#### 2.3.2 Behavioral Procedures

##### 2.3.2.1 Experiment 1: Standardization of VPA Model

**FIGURE 1:**
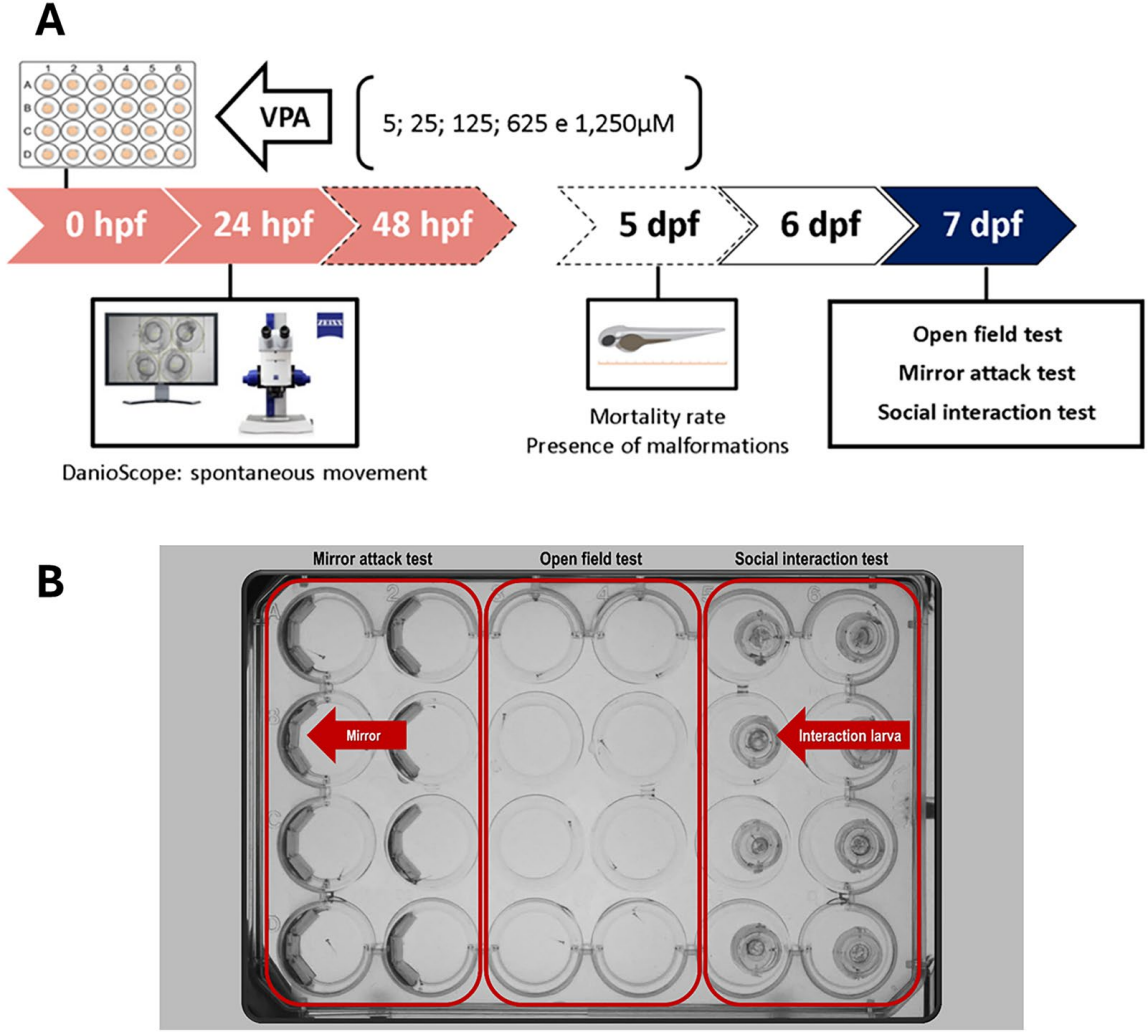
Experimental design of Experiment 1. (A) Schematic representation of the VPA's embryonic exposure model. Pink arrows: duration of the VPA stimulus in embryos. White arrows: dashed lines indicate the day of measurements for mortality rates and malformations. Solid line: larvae were left undisturbed in their home aquarium. Dark blue arrows: behavioral experiments. hpf: hours post‐fertilization; dpf: days post‐fertilization. (B) Adapted 24‐well plate (8 wells for the open field test, 8 wells for the mirror attack test, and 8 wells for the social interaction test) used for behavioral assessments at the larval stage 7 days post‐fertilization (dpf).

Experiment 1 aimed to determine the optimal working concentration of VPA that reliably induces neurodevelopmental changes in a zebrafish model for ASD without causing teratogenicity or lethality. After the embryonic VPA exposure (Figure 1A, see Section 2.3.1 for details), on the 7th day post‐fertilization (dpf), independent groups of zebrafish larvae were subjected to the following behavioral tests: open field (for motor activity and swimming coordination), the mirror attack test (for assessing aggressive behavior), and the social interaction (SI) test (to measure social behavior) (Figure 1B). At the end of the analysis, the larvae were euthanized under deep anesthesia.

##### 2.3.2.2 Experiment 2: Characterization of theBehavioral Effects of Different CBD Concentrations in the Zebrafish Model of VPA‐Induced Behavioral Changes Following Embryonic Exposure

**FIGURE 2:**
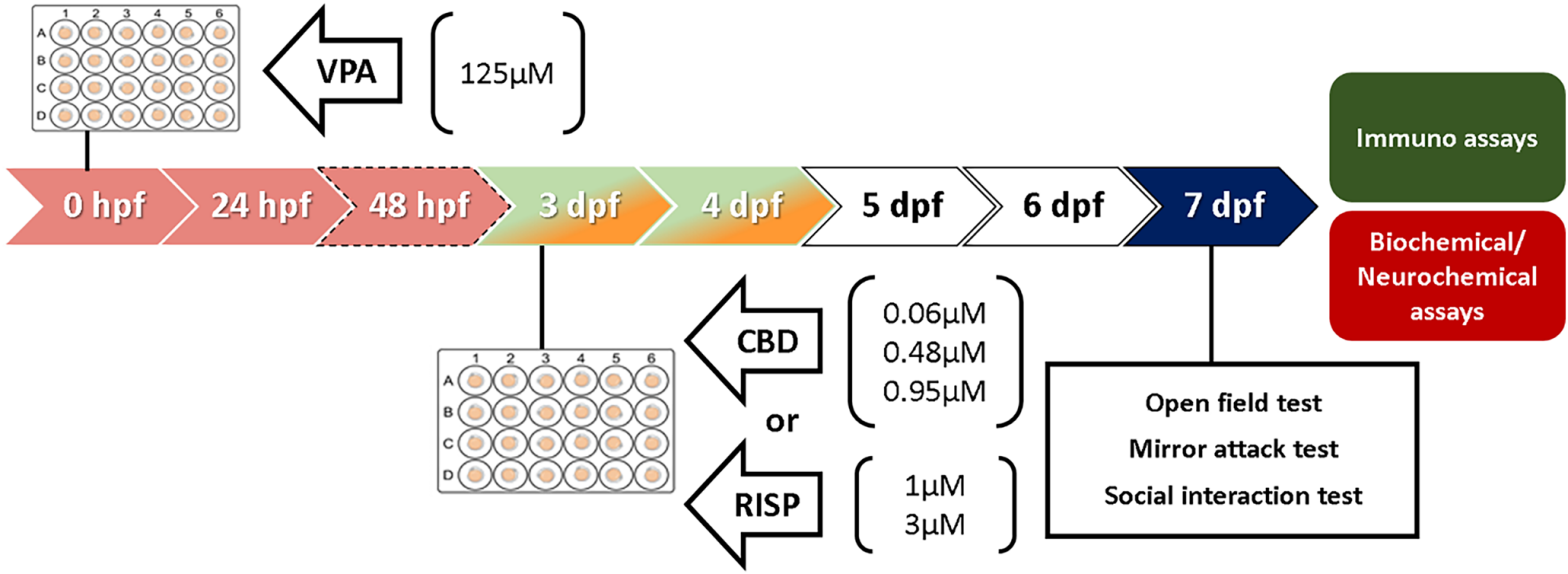
Experimental design of Experiment 2. Similar to Experiment 1, however, the green/yellow arrows represent the duration of the pharmacological treatments (CBD or RISP), while the white arrows indicate the washout period before the behavioral tests (blue arrow), and the immunofluorescence/biochemical/neurochemical assays. For further details, see the caption for Figure 1.

Once the VPA model was set (work concentration of 125 μM), we tested if the treatments could reverse the behavioral effects of VPA embryonic exposure (first 48 hpf). Then, on 3–4 dpf, larvae were exposed to different concentrations of vehicle (negative control), CBD, or RISP (positive control) (Figure 2). After a 2‐day drug washout period, independent groups of larvae were subjected to behavioral tests (for further details, see Figure 2 caption). At the end of the experiments, the larvae were euthanized and prepared for biochemical assays, neurotransmitter measurements, or immunofluorescence protocols (for further details, see Figure 2).

#### 2.3.3 Behavioral Tests

All behavioral tests were conducted in triplicate during the dark cycle (*n* = 20–24 larvae per group).

##### 2.3.3.1 Open Field Test (OFT)

Each larva was individually transferred to a new well in a modified 4–24‐well plate (Figure 1B), filled with 2 mL of tank water. The larvae were given 15 min to freely explore the well, consisting of a 5‐min habituation period followed by a 10‐min testing phase. The total distance traveled during the final 10 min of the test was recorded and analyzed automatically using the Zebrabox (View Point Life Sciences, France) (Dwivedi et al. 2019).

##### 2.3.3.2 Mirror Attack Test

This test was used to assess aggressive behavior. The wells of the modified 4–24‐well plate were filled with 2 mL of tank water and had half of their walls lined with a semicircular mirror (Figure 1B), designed to simulate the presence of another fish in the well. The larvae were allowed to freely explore the well with the first 5 min serving as a habituation period, followed by a 10‐min test. During the test, the number of attacks/bites directed toward the mirror and the total time spent in the mirror area were recorded and analyzed (Pham et al. 2012).

##### 2.3.3.3 SI Test

SI was assessed by placing a larva in a modified 4–24‐well plate filled with 2 mL of tank water. The well was divided into two semicircles, each with a diameter of 6.6 mm, using an adapted transparent slide that allowed each larva to visualize its counterpart in the adjacent semicircle (Figure 1B). The test consisted of a 5‐min habituation period, followed by a 10‐min testing phase. During the test, the time spent in the interaction zone, defined as the area of the well corresponding to a length, was analyzed automatically using the Zebrabox (View Point Life Sciences, France).

#### 2.3.4 Biochemical Assays

At the end of behavioral experiments, larvae were euthanized in liquid nitrogen (hypothermia) and stored at −80°C. Larval samples were subjected to ultrasonic homogenization with 1.5 mL of 0.1 M KPBS (pH 7.4). The homogenate was then equally divided into two aliquots: one designated for lipid peroxidation (LPO) analysis and the other for mitochondrial isolation. To isolate the mitochondria, the samples were centrifuged at 10,000 *g* for 20 min at 4°C, and catalase activity (CAT), glutathione‐S‐transferase (GST) activity, and protein quantification were assessed. All biochemical assays were conducted using a microplate spectrophotometer (Multiskan‐Sky) along with SkanIt software (version 6.0.2, Thermo Scientific, USA) to ensure accurate measurements and data analysis. The results of the biochemical assays were normalized to the total protein concentration, quantified by Bradford assay (Bradford 1976).

##### 2.3.4.1 GST Activity

The GST activity was continuously monitored for 30 min using a microplate spectrophotometer set to a wavelength of 340 nm by measuring the reaction between reduced glutathione and 1‐chloro‐2,4‐dinitrobenzene (CDNB). The enzymatic activity was normalized to the total protein content and expressed as nmol/min/mg of protein (Habig et al. 1974).

##### 2.3.4.2 CAT

The enzymatic CAT was continuously monitored for 2 min by measuring the decrease in absorbance at a wavelength of 240 nm, corresponding to the consumption of hydrogen peroxide (H_2_O_2_). Similar to the procedure for GST, the CAT was normalized to the total protein content and expressed in appropriate units (Claiborne 1985).

##### 2.3.4.3 Lipid Oxidation Quantification

For the LPO assay, the homogenate was treated with 2,6‐di‐tert‐butyl‐4‐methylphenol at a concentration of 4% (v/v), diluted in methanol, to prevent further oxidation. The endogenous LPO products were quantified as reactive substances reacting with thiobarbituric acid (TBARS), expressed as pmol per larva. The analysis was conducted at a wavelength of 535 nm (Bird and Draper 1984; Ohkawa et al. 1979) and normalized by total protein.

#### 2.3.5 Neurochemical Analyses by ELISA

To assess the levels of serotonin (5‐HT), dopamine (DA), anandamide (AEA), and 2‐arachidonoylglycerol (2‐AG) in the larvae, three clusters of 30 zebrafish larvae at 7 dpf were collected for each experimental group (*n* = 3 replicates per pool of 30 larvae). Quantifications were performed using commercial ELISA: Serotonin/5‐Hydroxytryptamine (ST/5‐HT) ELISA Kit (Cat. No. MBS2513872), Fish Dopamine (DA) ELISA Kit (Cat. No. MBS912178), General Anandamide (AEA) ELISA Kit (Cat. No. MBS2097802), and General 2‐Arachidonoylglycerol ELISA Kit (Cat. No. MBS2031975), following the manufacturer's instructions (kits from MyBioSource, USA).

#### 2.3.6 Immunofluorescence Assay and Microscopy

Intact larvae from behavioral assays were euthanized by hypothermia and fixed overnight at 4°C in 4% paraformaldehyde (PFA) with gentle agitation (300 rpm). The next day, samples were washed three times for 5 min each in PBS (0.01 M, pH 7.4), then dehydrated through graded methanol (25%, 50%, 75%, 100%, 5 min each) and stored at −20°C in 100% methanol. For the staining procedure, larvae were washed three times (5 min each) in 1% PBS‐Triton and gradually rehydrated in 75%, 50%, and 25% methanol in PBS (5 min each), followed by three additional washes in 1% PBS‐Triton. Depigmentation was performed by incubating larvae for 5 min in 3% hydrogen peroxide and 1% potassium hydroxide in PBS. Antigen retrieval was carried out with Proteinase K (10 μg/mL) for 40 min, followed by three washes in 1% PBS‐Triton. The blocking step was performed for 2 h at room temperature in 5% fetal bovine serum, 4 mg/mL bovine serum albumin, 1% Triton X‐100, and 1% DMSO in PBS. Larvae were then incubated overnight at 4°C with primary antibodies diluted in blocking solution (see Table S1 in Supporting Information), followed by six 30‐min washes in 1% PBS‐Triton. Secondary antibody incubation was performed overnight at 4°C under gentle agitation (see Table S1 in Supporting Information). The next day, larvae were incubated for 1 h in Hoechst (1:10,000, Invitrogen) diluted in 1% PBS‐Triton. After the final washes, larvae were mounted on glass slides using Fluoromount‐G (Invitrogen) and coverslipped. Imaging was performed using multiphoton microscopy (LSM 780 Axio Observer, Carl Zeiss) at 40× magnification (MOSAIC mode), with images acquired in z‐stacks and analyzed with Zen 3.5 software (Carl Zeiss). Quantification was conducted on the total brain area, and results were expressed as relative fluorescence intensity (FI) for each immunostaining (*n* = 2–6 larvae/group).

### 2.4 Statistical Analyses

When the assumptions of normality and homoscedasticity were met, significant differences between treatments were assessed using one‐way ANOVA or two‐way ANOVA, followed by Duncan's post hoc test. Data are presented as the mean ± standard error of the mean (SEM). For nonparametric data, significant differences were evaluated using the Kruskal–Wallis test, followed by Dunn's post hoc test, with results expressed as median and interquartile ranges. Statistical significance was defined as *p* ≤ 0.05.

## 3 Results

### 3.1 Embryonic Exposure to VPA in Zebrafish Induces Long‐Term Behavioral Changes Associated With ASD

**FIGURE 3:**
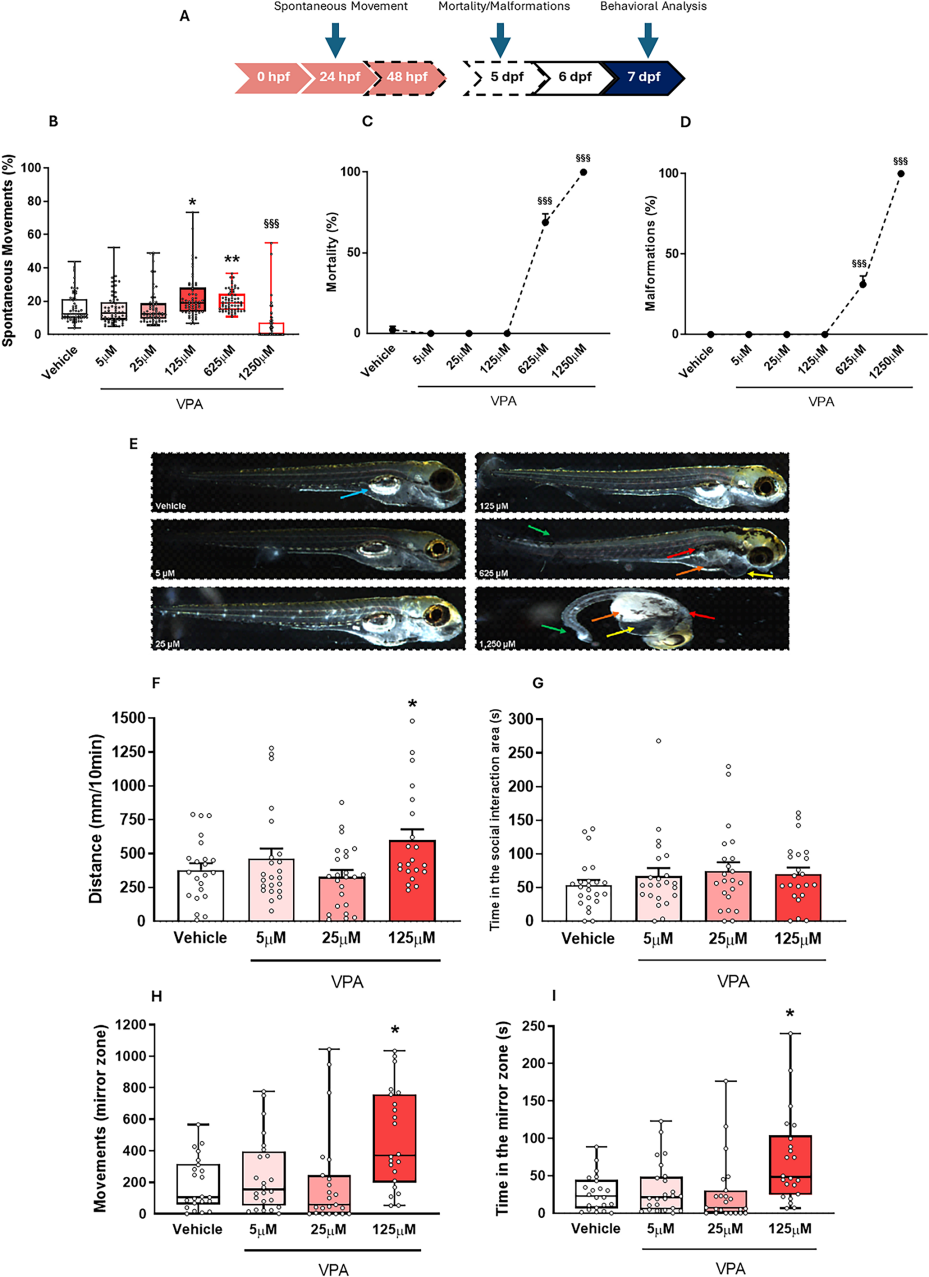
Acute and long‐term consequences of embryonic exposure to different concentrations of VPA. (A) Timeline of the analysis. (B) VPA exposure (pink arrow) changes the percentage of movement after 24 h. (C and D) Zebrafish exhibited increased rates of malformations and mortality by 5 days post‐fertilization (dpf) depending on the concentration of VPA. (E) Representative images of observed malformations: blue arrows indicate inflated swim bladders, red arrows denote non‐inflated swim bladders, orange arrows show yolk sac edema, yellow arrows highlight pericardial edema, and green arrows point to tail malformations. Data are presented as mean ± standard error of the mean (SEM), with *n* = 61–63 larvae per group. (F–I) Behavioral analysis of the long‐term (7 dpf, blue arrow) behavioral effects of different consequences of VPA embryonic exposure: (F) open field test—represented as mean ± SEM, with *n* = 21–24 larvae per group; (G) social interaction test—represented as mean ± SEM, *n* = 22–23 larvae per group; (H) the number of movements toward the mirror was recorded; (I) the time spent in the mirror area was also measured. Data are shown as median and interquartile ranges, with *n* = 22–24 larvae per group. Statistical analyses were performed as follows: Kruskal–Wallis test with Dunn's post hoc test for panels (B, H, I); one‐way ANOVA with Duncan's post hoc test for panels (C, D, F, G). Significance levels are indicated as: *p < 0.05, **p < 0.01 versus the vehicle group; ^§§§^ *p* < 0.001 versus all groups. For a detailed description of the experimental design, please see Figure 1.

In rodents, embryonic VPA exposure induces social deficits, altered exploration, impaired motor coordination, and aggression. To investigate if similar patterns occur in zebrafish, we characterized the effects of VPA during early development by continuous observation from early stages to larvae in the same individuals, a clear experimental advantage over the rodent model. Exposure to different VPA concentrations (5, 25, 125, 625, 1250 μM) for 24 h significantly altered tail coiling behavior (*χ* ^2^ = 125.81; *p* < 0.001, Figure 3B). Specifically, 125 μM (*p* < 0.05) and 625 μM (*p* < 0.01) increased spontaneous movement, while 1250 μM of VPA reduced this behavior (*p* < 0.001). Higher VPA concentrations (625 and 1250 μM) also increased malformations (*F*_5,12_ = 346.12; *p* < 0.001, Figure 3D) and mortality rates (*F*_5,12_ = 358.53; *p* < 0.001, Figure 3C), with 100% lethality at the concentration of 1250 μM. The VPA concentrations of 5, 25, and 125 μM did not induce malformation and mortality (Figure 3C,D) throughout the experimental window, and therefore, were selected to continue the experiments.

To assess the long‐term effects of non‐teratogenic concentrations of VPA (5, 25, 125 μM), we tested 7 dpf larvae in behavioral paradigms: mirror attack (aggression), SI, and open field (locomotor activity) (Figure 3F–I). In the OFT, 125 μM VPA larvae showed increased locomotor activity compared to controls (*F*_3,86_ = 3.42; *p* < 0.05, Figure 3F). Larvae exposed to 125 μM VPA also exhibited increased movements (*χ* ^2^ = 16.02; *p* < 0.01, Figure 3H) and time (*χ* ^2^ = 15.82; *p* < 0.01, Figure 3I) in the mirror area, indicating increased aggressive behavior. No significant differences were found in SI (*F*_3,86_ = 0.71; *p* > 0.05, Figure 3G). Based on these results, 125 μM was selected for subsequent experiments for testing drug efficacy.

### 3.2 CBD is More Potent and Safer Than RISP in Reversing VPA Embryonic Exposure‐Induced ASD‐Like Behaviors in Zebrafish Larvae

RISP is commonly prescribed for managing ASD symptoms. Therefore, as a positive control for our model, we tested whether RISP (1 and 3 μM) could counteract behavioral changes induced by embryonic VPA (125 μM) exposure in zebrafish larvae. Our results suggest that RISP at a concentration of 1 μM effectively reversed ASD‐like behaviors (at 7 dpf) induced by VPA exposure (Figure S1 in Supporting Information).

**FIGURE 4:**
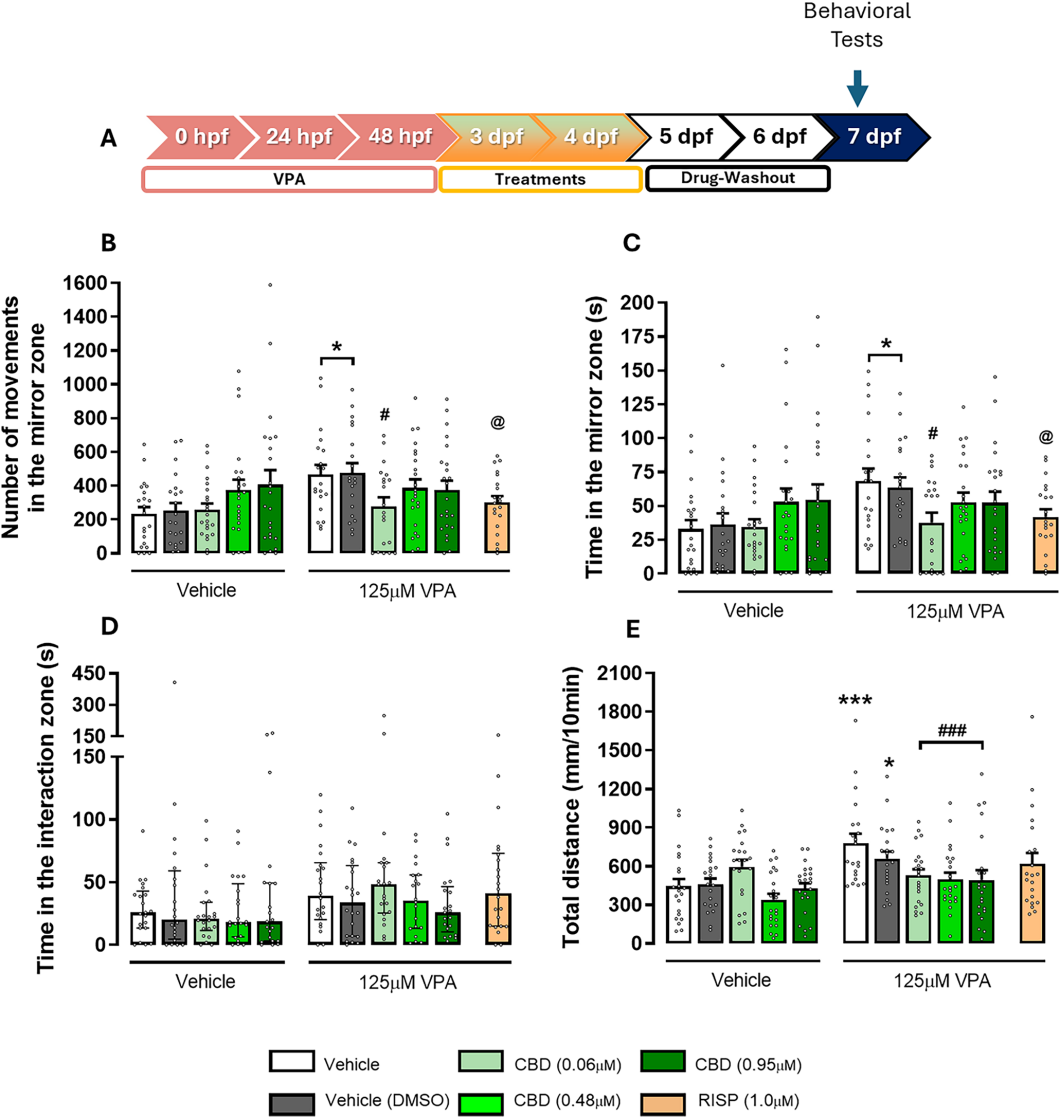
Cannabidiol (CBD) is more potent than risperidone (RISP) in reverting the behavioral effects of embryonic sodium valproate (VPA) exposure. (A) Timeline of the experiments. Independent groups of larvae were submitted to: (B) mirror attack test—number of movements in the mirror area reported as absolute values; (C) time spent in the mirror area in seconds, with *n* = 21–23 larvae per group presented as mean ± SEM; (D) social interaction test—time in the interaction area in seconds, with *n* = 20–22 larvae per group, presented as median and interquartile ranges; (E) open field test with *n* = 21–22 larvae per group presented as mean ± SEM. Statistical analyses: two‐way ANOVA followed by Duncan's post hoc test, and one‐way ANOVA followed by Duncan's post hoc test. Results are reported as follows: *p < 0.05, ***p < 0.001 (compared to the vehicle group); ^#^ *p* < 0.05, ^###^ *p* < 0.001 (compared to the 125 μM VPA/vehicle group); and ^@^ *p* < 0.05 (compared to the 125 μM VPA/vehicle and 125 μM VPA/DMSO groups). For a detailed description of the experimental design, please see Figure 2.

Next, given CBD's broad action in neurodevelopmental models, we evaluated CBD at 0.06, 0.48, and 0.95 μM alongside a vehicle (0.01% DMSO) and RISP (1 μM, Figure 4A). In the mirror attack test, both VPA‐exposed groups showed an increased number of movements (*F*_1,205_ = 6.51; *p* < 0.05) and time spent in the mirror area (*F*_1,205_ = 5.78; *p* < 0.05) compared to vehicle‐treated groups (Figure 4B,C). Treatment with CBD (0.06 μM) or RISP (1 μM) significantly reduced these parameters compared to untreated VPA groups (since CBD and RISP have different vehicles—DMSO or saline, respectively) (*F*_5,120_ = 2.27; *p* < 0.05). No significant difference between CBD and RISP was detected (Figure 4B,C).

To determine if any CBD concentrations affect locomotor activity in 7 dpf zebrafish larvae exposed to 125 μM VPA during the embryonic period, larvae were tested in the OFT (Figure 4E). Both VPA‐exposed non‐treated groups (saline or 0.01% DMSO) had significantly higher locomotor activity than the vehicle control group (*F*_4,205_ = 3.58; *p* < 0.01). In contrast, VPA‐exposed larvae treated with all the CBD concentrations showed a significant reduction in total distance compared to the untreated VPA group (*p* < 0.001, Figure 4E). No effects on the SI test were observed (*χ* ^2^ = 12.77, Figure 4D).

During the collection of larval samples in plastic vials filled with PBS following euthanasia, we made an interesting observation: the larvae treated with RISP (independent of the stimulus) tended to settle at the bottom of the vial, which was distinctly different from the behavior of larvae in the vehicle and CBD treatment groups. A close morphological analysis revealed that RISP induced notable changes in the swim bladder development in these larvae (Figure S2 in Supporting Information).

Given that CBD and RISP reversed the behavioral effects induced by VPA at different concentrations (0.06 μM for CBD and 1 μM for RISP), we conclude that, in this model, CBD is more potent than RISP in reversing VPA‐induced ASD‐like behaviors in zebrafish larvae at 7 dpf. Additionally, given the close relationship between the swim bladder and the primitive lung, we propose that CBD exhibits a safer profile than RISP in our zebrafish model.

### 3.3 CBD Reduces LPO in Zebrafish: A Potential Mechanism Underlying Its Neuroprotective and Behavioral Effects

**FIGURE 5:**
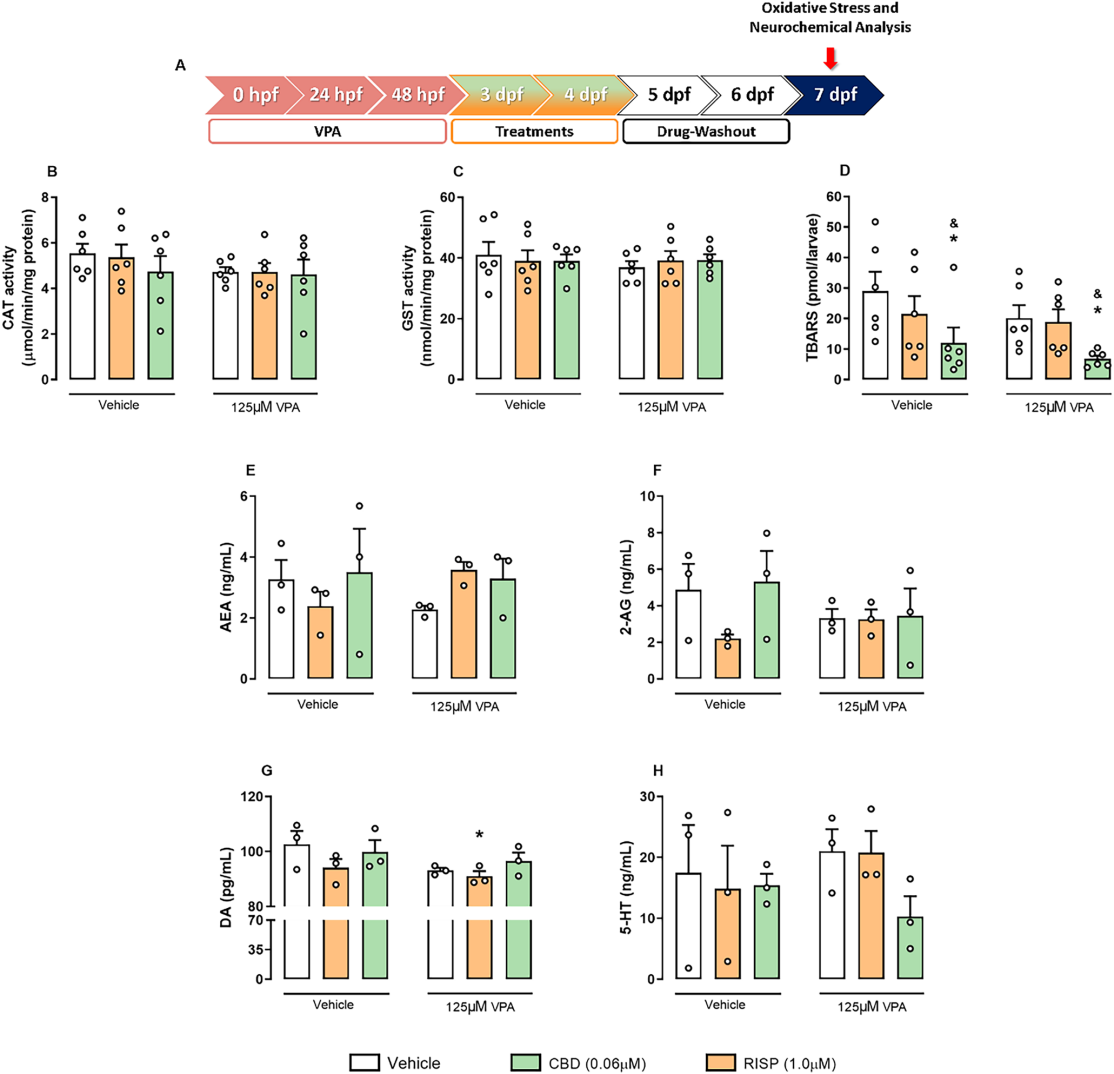
Oxidative stress and neurotransmitter levels measured panel in VPA model. (A) Schematic timeline of the assays in independent groups of larvae. (B) Catalase (CAT) activity. (C) Glutathione‐S‐transferase (GST). (D) Lipid peroxidation products, quantified as thiobarbituric acid reactive substances (TBARS) (*n* = 6 pools of 30 larvae/group; represented as mean ± SEM). (E) Total anandamide (AEA) and (F) 2‐AG levels. (G) Total dopamine (DA) and (H) total serotonin (5‐HT) levels are expressed in ng/mL. Data from triplicates (*n* = 3 pools of 30 larvae/group; represented as mean ± SEM). Two‐way ANOVA followed by Duncan's post hoc test (*p < 0.05 compared to the vehicle group). One‐way ANOVA followed by Duncan's post hoc test (^&^ *p* < 0.05 compared to the other groups, regardless of VPA exposure).

Although observational studies suggest benefits of CBD on ASD symptoms, its molecular mechanisms remain unclear. To explore this, we analyzed the antioxidant system and neurochemical profiles in zebrafish brains using the lowest effective CBD concentration (0.06 μM) and compared its effects with RISP (Figure 5A). Analysis of CAT (Figure 5B) and GST activity (Figure 5C) showed no significant changes after VPA exposure or RISP/CBD treatments. Conversely, LPO levels, assessed via TBARS, were significantly reduced by CBD treatment, indicating a protective antioxidant effect, regardless of VPA exposure (*F*_2,30_ = 5.33; *p* < 0.05, Figure 5D).

We also evaluated the levels of the neurotransmitters, DA and 5‐HT, and the endocannabinoids, 2‐AG and AEA. Although VPA treatment showed a graphical trend in reducing AEA (*F*_2,12_ = 2.8; *p* = 0.10, Figure 5E) and 2‐AG (*F*_2,12_ = 1.01; *p* > 0.05, Figure 5F) levels, they remained unchanged across groups. DA levels (*F*_1,12_ = 3.09; *p* > 0.05, Figure 5G) were significantly reduced in VPA‐exposed larvae treated with RISP compared to controls (*p* < 0.05). However, 5‐HT levels (*F*_2,12_ = 0.66; *p* > 0.05, Figure 5H) showed no significant changes after VPA exposure or RISP/CBD treatment.

### 3.4 The Embryonic Exposure to VPA Induces Complex Changes in the Neurodevelopment Markers in Zebrafish Larvae: CBD'S and RISP'S Effects

**FIGURE 6:**
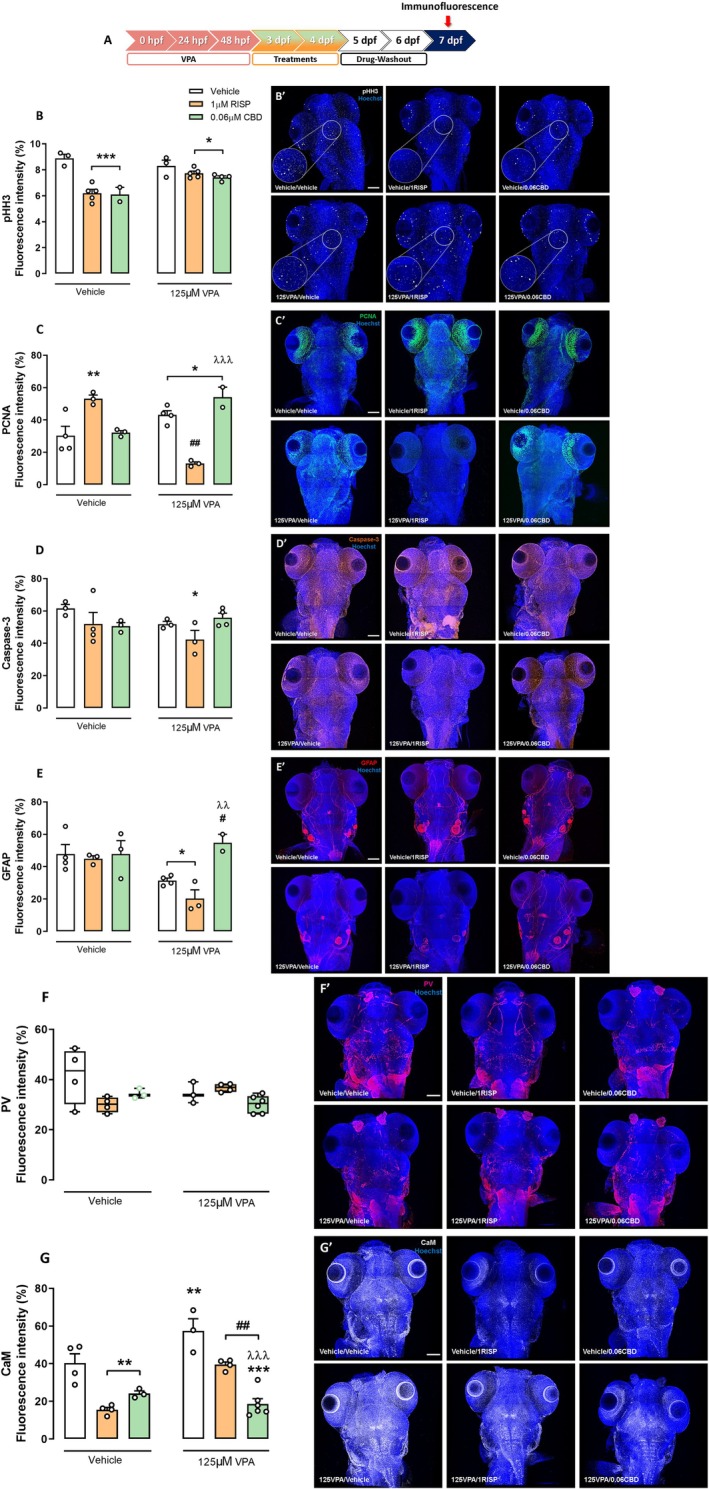
Neurodevelopment markers analyzed 7 days after embryonic VPA exposure in zebrafish treated with vehicle, RISP, or CBD. (A) Timeline of the experiments. (B–G) % of fluorescence intensity (FI) for (B) pHH3, (C) PCNA, (D) Caspase‐3, (E) GFAP, (F) PV, and (G) CaM quantification in the brain of 7 dpf zebrafish larvae exposed to 125 μM VPA during the embryonic period and treated with 1 μM RISP or 0.06 μM CBD concentrations. (B–E and G) Data in triplicate (*n* = 2–6 larvae/group) expressed as mean ± SEM of percentage of FI (%). Two‐way ANOVA followed by Duncan's post hoc test. *p < 0.05, **p < 0.01, and ***p < 0.001 compared to the vehicle group; ^#^ *p* < 0.05 and ^##^ *p* < 0.01 compared to the 125 μM VPA/vehicle group; ^λλ^ *p* < 0.01 and ^λλλ^ *p* < 0.001 compared to the 125 μM VPA/1 μM RISP group. (F) Data in triplicate (*n* = 3–6 larvae/group) expressed as median and interquartile ranges of percentage of FI (%). Kruskal–Wallis followed by Dunn's test for multiple comparisons. Representative Z‐stack images‐MOSAIC for (B′) pHH3, (C′) PCNA, (D′) Caspase‐3, (E') GFAP, (F′) PV, and (G') CaM obtained by multiphoton microscopy (40×); scale = 100 μm.

Neurodevelopment relies on fine‐tuned cellular regulation, and any disruptive processes may contribute to ASD (Satterstrom et al. 2020). Here, we evaluated key neurodevelopmental markers at 7 dpf, including proliferation (pHH3, PCNA) and apoptosis (Caspase‐3) markers, GFAP expression (astrocyte, radial glial, and possibly neural stem cells), and the calcium signaling proteins, CaM and PV (Figure 6A).

For pHH3, both VPA‐exposed and control larvae treated with 1 μM RISP or 0.06 μM CBD showed a significant reduction in FI compared to controls (*F*_2,16_ = 21.29; *p* < 0.001; Figure 6B). VPA exposure tended to increase PCNA expression (Figure 6C), with 1 μM RISP increasing PCNA in non‐VPA larvae, but decreasing it in VPA‐exposed ones (*F*_2,13_ = 35.60; *p* < 0.001). CBD increased PCNA in VPA‐exposed larvae (*p* < 0.05). RISP reduced Caspase‐3 expression in VPA‐exposed larvae (*p* < 0.05; Figure 6D). GFAP analysis revealed reduced expression in VPA‐exposed larvae treated with vehicle or RISP compared to controls (*F*_1,13_ = 6.95; *p* < 0.05). CBD restored GFAP levels compared to VPA‐non‐treated (*p* < 0.05) and VPA‐RISP‐treated groups (*p* < 0.01) (Figure 6F).

The calcium signaling/binding proteins (CAM and PV) analyses revealed that VPA embryonic exposure increased CaM expression at 7 dpf, which was reversed by RISP and CBD treatment after drug washout (*F*_2,18_ = 10.17; *p* < 0.01; Figure 6G). Both drugs reduced CaM in controls. No significant changes were detected for PV expression (*χ* ^2^ = 10.93; *p* > 0.05; Figure 6F). These findings suggest that CBD corrects the excitatory imbalance and astrocyte dysfunction caused by VPA exposure.

## 4 Discussion

Our study, in line with previous work (for an extensive review, please read Camussi et al. 2025), demonstrates that embryonic exposure of zebrafish to 125 μM VPA produces long‐term behavioral phenotypes consistent with ASD, validating a fast and reproducible model for rapid drug screening in ASD research. Treatment with the lowest evaluated concentration of CBD (0.06 μM) effectively reversed these behavioral changes, proving to be more potent and safer than RISP (1 μM), a standard treatment for ASD. Importantly, we also begin to uncover a possible mechanistic basis for CBD's effects, linked to reductions in LPO and changes in markers of neurodevelopment.

The VPA exposure during early zebrafish development replicates several core and associated behavioral and neurochemical features of ASD, consistent with findings in rodent models (Kim et al. 2011; Schneider and Przewłocki 2005). Zebrafish embryos exposed to VPA showed dose‐dependent changes in spontaneous tail coiling, increased malformation rates (e.g., non‐inflated swim bladders and edema), and heightened mortality at higher concentrations. These results align with previous studies using VPA to model ASD‐like symptoms, such as hyperactivity and aggression (Chen et al. 2020; Christensen et al. 2013; Meshalkina et al. 2018). An advantage of the zebrafish model compared to rodents is the ability to observe the embryonic effects of VPA in real time, thanks to external development and egg transparency. Moreover, zebrafish embryos hatch within hours, whereas rodent gestation requires approximately 21 days. Embryos exposed to 125 μM VPA from 4 to 48 hpf exhibited persistent hyperactivity and aggression even after exposure cessation, suggesting long‐lasting neurodevelopmental consequences. Notably, this exposure did not affect survival, malformations, or hatching time, but led to increased spontaneous movement, motor activity, and aggressive‐like behavior at 7 dpf (Dwivedi et al. 2019; Zimmermann et al. 2015). These findings support the well‐documented teratogenic and neurodevelopmental impacts of VPA in humans and rodents (Choi et al. 2016; Ornoy et al. 2023) and further validate the zebrafish as a sensitive and translational model for studying early developmental disturbances relevant to ASD.

Our model also reproduces the long‐term behavioral effects of VPA. Larvae exposed to 125 μM VPA showed increased aggressive behavior, as evidenced by enhanced mirror attack responses, and increased locomotor activity in the OFT—two phenotypes previously associated with hyperactivity, impulsivity, and social dysregulation (Mabunga et al. 2015). Interestingly, no significant changes were observed in the SI test at 7 dpf, suggesting that deficits in social preference may emerge later in development or that our assay conditions lacked the sensitivity to detect subtle impairments at this early stage in the zebrafish larva. The absence of VPA's effects on SI may be associated with the immature state of social behavior regulation at this developmental point in zebrafish larvae (Buske and Gerlai 2012). However, previous studies suggested that the mirror attack test also has a good face of validity while studying social behaviors (Desjardins and Fernald 2010; Moretz et al. 2007). Together, these results indicate that early exposure to 125 μM VPA reliably induces ASD‐relevant phenotypes without compromising overall survival or development, supporting its use as an optimal concentration for further pharmacological testing.

Although a few studies have reported the protective effects of CBD in rodent models of ASD, and on certain neuropsychiatric symptoms in humans, to the best of our knowledge, CBD has not been tested in ASD models that include positive control groups, such as treatment with antipsychotic drugs (Pedrazzi et al. 2025; Silva Jr et al. 2024). When comparing therapeutic interventions, we found that CBD at a low concentration (0.06 μM) was more effective than RISP (1 μM) in reversing VPA‐induced aggressive behavior and hyperlocomotion. Importantly, larvae treated with RISP displayed swim bladder abnormalities, suggesting potential developmental side effects—a finding consistent with prior reports of RISP's adverse impacts on growth and organogenesis (Fukushima et al. 2023). Given that individuals with ASD often have communication difficulties or swallowing dysfunctions, they may be at a higher risk of aspiration pneumonia—a complication previously linked to RISP treatment (Wang et al. 2020; Dzahini et al. 2018). In contrast, CBD treatment was not associated with any apparent developmental toxicity, underscoring its safer profile in comparison to RISP.

Mechanistically, our biochemical assays revealed that neither VPA exposure nor treatments with RISP or CBD significantly altered some parameters of oxidative stress (CAT or GST) activities, indicating that these classical antioxidant defenses were not profoundly engaged in our model. However, CBD uniquely reduced the TBARS levels, suggesting a protective effect against LPO. Zebrafish larvae treated with CBD exhibited significantly lower TBARS levels than untreated and RISP‐treated groups. LPO has been implicated in the pathophysiology of ASD (Chauhan and Chauhan 2006; Rose et al. 2012), and its reduction by CBD provides a plausible mechanistic link to its behavioral benefits (Banji et al. 2011). CBD's antioxidant properties have been characterized across various neuroprotective contexts (Atalay et al. 2019; Campos et al. 2016; Hampson et al. 1998). At the neurochemical level, CBD did not change DA, 5‐HT, or endocannabinoid levels. Preliminary evidence suggests that CBD modulates endocannabinoid tone, including AEA levels (Campos et al. 2013), which could contribute to restoring the neurochemical balance disrupted by early VPA exposure.

Neurobiologically, GFAP staining initially suggested reduced astrocyte activity in VPA‐exposed animals. CBD treatment restored GFAP expression levels, suggesting a protective effect on glial populations or neurogenic niches disrupted by VPA, as it is important to emphasize that GFAP is also expressed in radial glial cells and neural stem cells during development and in postnatal brains (Alvarez‐Buylla et al. 2001), complicating straightforward interpretation. CBD can also facilitate postnatal neurogenesis (Campos et al. 2013). Additionally, we found that VPA increased the expression of the proliferating cell nuclear antigen (PCNA). RISP treatment significantly reduced this VPA‐induced PCNA upregulation, whereas CBD did not exert a significant effect. These findings may reflect a differential impact of treatments on neurogenesis or gliogenesis processes following early brain injury. Increased PCNA could indicate compensatory neurogenesis or reactive gliosis triggered by VPA (Sharma et al. 2018). The fact that CBD did not reduce PCNA levels suggests that, unlike RISP, CBD may allow ongoing repair or adaptation processes without suppressing endogenous proliferative responses. This could confer an advantage in preserving neural plasticity mechanisms essential for functional recovery in ASD models. Regarding calcium signaling and molecules that influence synapse formation and brain development (Bryan 2010), CaM expression was found elevated in VPA‐exposed animals, but reduced by both RISP and CBD, with CBD producing a more significant normalization. This result could reflect a partial reversal of the disrupted excitatory/inhibitory imbalance, which is considered a hallmark of ASD neuropathology (Nelson and Valakh 2015).

Overall, these findings suggest that CBD not only reverses key behavioral impairments associated with embryonic VPA exposure, but also does so with greater potency and a better safety profile than RISP. Given the translational value of the zebrafish model—offering rapid, high‐throughput behavioral and biochemical screening—our work provides a promising platform for further investigating CBD's therapeutic potential in ASD and related neurodevelopmental disorders. Future studies should explore the long‐term impacts of CBD treatment beyond early larval stages and elucidate the broader molecular pathways through which CBD confers neuroprotection.

However, as in every preclinical study, our results have limitations that should be acknowledged. First, our behavioral assessments were restricted to early larval stages (7 dpf); thus, we could not evaluate the persistence of VPA‐induced deficits or the durability of CBD's therapeutic effects in “adolescent/adult” zebrafish. Second, while we identified a reduction in LPO as a potential mechanism underlying CBD's benefits, this study did not explore other neurobiological pathways, such as inflammatory cascades. Finally, although zebrafish models offer valuable translational insights, the clinical implications of our findings require further investigation in mammalian models and, ultimately, in human studies.

## Funding

This work was supported by Fundação de Amparo à Pesquisa do Estado de São Paulo (15/05551‐0, 17/24304‐0, 20/05416‐4, 14/50891‐1) and INCT 2014: Translational Medicine (465458/2014‐9).

## Conflicts of Interest

F.S.G., J.A.C., and J.E.C.H. are the coinventors of the patent “Fluorinated CBD compounds, compositions, and uses thereof, Pub. No.: WO/2014/108899, International Application No.: PCT/IL2014/050023,” Def. US number Reg. 62193296; July 29, 2015; INPI on August 19, 2015 (BR1120150164927; Mechoulam R, Zuardi AW, Kapczinski F, Hallak JEC, Guimarães FS, Crippa JAS, Breuer A). Universidade de São Paulo (USP) has licensed this patent to Phytecs Pharm (USP Resolution No. 15.1.130002.1.1). A.C.C., F.S.G., J.A.C., and J.E.C.H. have an agreement with Prati‐Donaduzzi to “develop a pharmaceutical product containing synthetic CBD and prove its safety and therapeutic efficacy in the treatment of epilepsy, schizophrenia, Parkinson's disease, and anxiety disorders.” A.C.C., F.S.G., J.A.C., and J.E.C.H. are the coinventors of the patent “Cannabinoid‐containing oral pharmaceutical composition, method for preparing and using the same,” INPI on September 16, 2016 (BR 112018005423‐2). J.A.C. was a consultant and/or received speaker fees and/or sits on the advisory board and/or receives research funding and/or receives speaker fees from Janssen‐Cilag, Torrent Pharm, Ease Labs Pharm, Prati‐Donaduzzi, Mantecorp, ArtMed, PurMed Global, BioBrains, and BSPG Pharm over the past 5 years. J.A.C. is a member of the International Advisory Board of the Australian Centre for Cannabinoid Clinical and Research Excellence (ACRE)—National Health and Medical Research Council (NHMRC). The remaining authors declare no conflicts of interest.

## Supporting information


**Data S1:** Supporting information.

## Data Availability

The data that support the findings of this study are available from the corresponding author upon reasonable request.
