# Supplementary material for: Comparative Analysis of Cannabidiol and Risperidone on Behavioral and Neurochemical Outcomes, and Neurodevelopment Markers in a Zebrafish Model of Embryonic Exposure to Sodium Valproate
Source: Autism Res. 2025 Nov 26;18(12):2368–81. doi: 10.1002/aur.70151 (PMC12729503; doi:10.1002/aur.70151)
Supplement: Supplementary file 1 — Data S1: Supporting information. [file AUR-18-2368-s001.docx]

**Supplementary Material**

**Comparative analysis of cannabidiol and risperidone on behavioral and neurochemical outcomes, and neurodevelopment markers in a zebrafish model of autism spectrum disorder.**

Karla C. M. Costa^1^, Tamires A. V. Brigante^1,2^, Pedro H. C. Lirio^1,2^, Gabriel G. Fernandes^1^, Franciele F. Scarante^1^, Davi S. Scomparin^1,2^, Rafael R. Ferreira^1^; Maria A. Vicente^1,2^, Flavia R. Abe^3^, Francisco S. Guimarães^1,2,4^, Jaime E. C. Hallak^2,4,5^, Jose A. Crippa^2,4,5,6^, Danielle P. de Oliveira^3^, Alline C. Campos^1,2,4*^

^1^ Department of Pharmacology; Ribeirão Preto Medical School- University of São Paulo- Brazil.

^2^ Center for Cannabinoid Research-Mental Health Building- Ribeirão Preto Medical School, University of São Paulo- Brazil.

^3^ Department of Clinical Analyses, Toxicology and Food Science, School of Pharmaceutical Sciences of Ribeirão Preto, University of São Paulo, São Paulo-Brazil.

^4^ National Institute of Science and Technology for Translational Medicine (INCT TM) - CNPQ/FAPESP/CAPES - Brazil.

^5^ Department of Neuroscience and Behavior, Ribeirão Preto Medical School, University of São Paulo, São Paulo-Brazil.

^6^ Laboratory of Psychiatric Neuroimaging (LIM21), Hospital das Clinicas HCFMUSP, Faculdade de Medicina, Universidade de Sao Paulo, Sao Paulo, SP, BR

*Corresponding author

Alline C. Campos

Pharmacology of Neuroplasticity Lab, Department of Pharmacology, Ribeirão Preto Medical School- University of São Paulo; Av. Bandeirantes, 3900, Monte Alegre, Ribeirão Preto-SP-Brazil; CEP:14049900 (allinecampos@usp.br); Phone: +551633150217

**Material and Methods**

**Immunofluorescence:**

The list of primary and secondary antibodies used in the immunofluorescence protocols can be seen in the Supplementary Table 1.

**Supplementary Table 1:** List of antibodies used in immunofluorescence assays.

| **ANTIBODY** | **CATEGORY** | **HOST/TARGET** | **CAT.NUMBER/BRAND** | **DILUTION** |
| --- | --- | --- | --- | --- |
| **Histone H3** | Primary | Rabbit | ab183626/Abcam | 1:400 |
| **Caspase-3** | Primary | Rabbit | ab13847/Abcam | 1:400 |
| **GFAP** | Primary | Goat | ab53554/Abcam | 1:400 |
| **PCNA** | Primary | Rabbit | ab18197/Abcam | 1:400 |
| **Parvalbumin** | Primary | Mouse | SAB4200545/Sigma-Aldrich | 1:50 |
| **Calmodulin** | Primary | Rabbit | ab45689/Abcam | 1:120 |
| **Alexa Fluor 594nm** | Secondary | Goat anti-rabbit | Invitrogen | 1:500 |
| **Alexa Fluor 594nm** | Secondary | Goat anti-mouse | Invitrogen | 1:500 |
| **Alexa Fluor 594nm** | Secondary | Donkey anti-goat | Invitrogen | 1:500 |
| **Alexa Fluor 647nm** | Secondary | Goat anti-rabbit | Invitrogen | 1:500 |
| **Alexa Fluor 488nm** | Secondary | Donkey anti-rabbit | Invitrogen | 1:500 |

**Supplementary Results**

**Risperidone (RISP) reverses the behavioral effects of the VPA embryonic exposure.**

Analysis of the absolute number of movements in the mirror area (SFig. 1B) using the Two-way ANOVA test demonstrated a statistical effect of VPA exposure (F_1,127_=28.98; p<0.001) and did not demonstrate a statistical effect for the treatment with RISP (F_2,127_=0.56; p>0.05). However, the analysis returned the statistical interaction between the exposure × treatment variables (F_2,127_=5.37; p<0.01). Thus, it is possible to observe that animals in the Vehicle/1RISP group demonstrated a statistically significant increase in the number of movements in the mirror compared to the vehicle group (p<0.05). Furthermore, animals exposed to 125μM VPA and treated or not with different RISP concentrations (125VPA/Vehicle; 125VPA/1RISP and 125VPA/3RISP) showed a statistically significant increase in the absolute number of movements in the mirror compared to animals in the vehicle group (p<0.01). Also, animals in the 125VPA/1RISP group displayed a statistically significant reduction in the absolute number of movements in the mirror area compared to animals in the 125VPA/Vehicle group (p<0.05).

Regarding the time spent in the mirror area (SFig. 1C), analysis using the Two-way ANOVA test demonstrated a statistical effect of VPA exposure (F_1,127_=33.08; p<0.001) and did not demonstrate a statistical effect for treatment with RISP (F_2,127_=0.19; p>0.05). However, the analysis returned the statistical interaction between the exposure × treatment variables (F_2,127_=4.11; p<0.05). Thereby, it is possible to observe that animals exposed to 125μM VPA and treated or not with different RISP concentrations (125VPA/Vehicle; 125VPA/1RISP, and 125VPA/3RISP) showed a statistically significant increase in time in the mirror area compared to animals in the vehicle group (p<0.01). Furthermore, animals in the 125VPA/1RISP group showed a statistically significant reduction in the time spent in the mirror area compared to animals in the 125VPA/Vehicle group (p<0.05)**.**

In the Open Field test (SFig. 1D), statistical analysis of the total distance using the Kruskal-Wallis test demonstrated differences between the groups (χ^2^=26.40; p<0.001), making it possible to observe that animals exposed to 125μM VPA and without treatment (125VPA/Vehicle) displayed a statistically significant increase in the motor profile compared to animals in the vehicle group (p<0.05). None of the tested RISP concentrations could sustainably reverse this change in the motor profile. VPA exposure and RISP treatment did not induce changes in the social interaction profile of zebrafish larvae at the evaluated age (SFig. 1E).

**
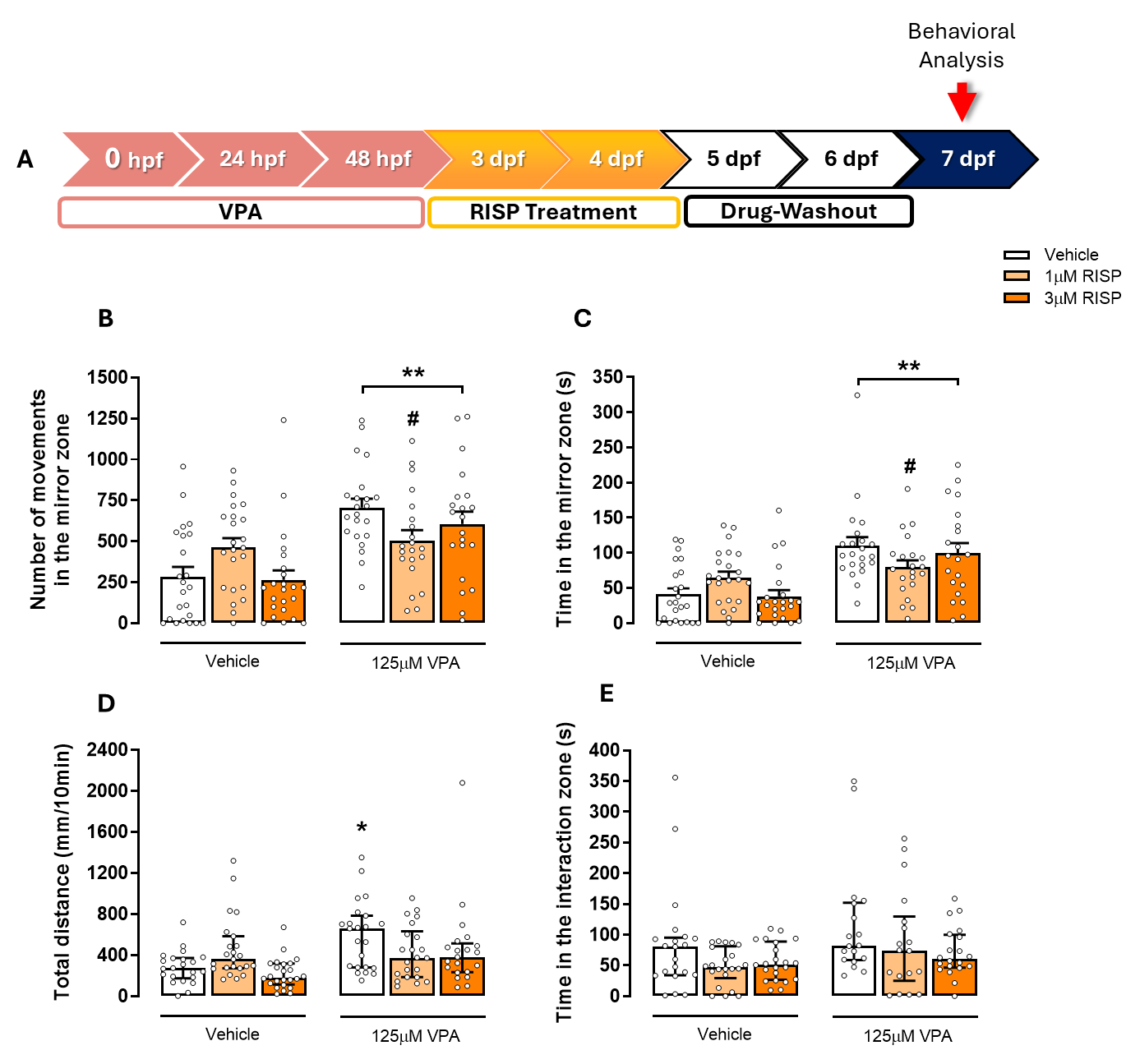
**

**S Figure 1:** (A)- Schematic representation of the experimental Design. (B and C): Mirror attack test for the aggressiveness profile assessment of 7 dpf zebrafish larvae exposed to 125μM VPA during the embryonic period and treated with different RISP concentrations. (B) Number of movements in the mirror area expressed in absolute values; (C) Time in the mirror area expressed in seconds (s). Triplicate data (n=21-23 larvae/group) expressed as mean ± SEM. Two-way ANOVA followed by Duncan's post hoc test. *p<0.05 and **p<0.01 compared to the vehicle group; ^#^p<0.05 compared to the 125VPA/Vehicle group. (D) Open field test for motor activity analysis of 7 dpf zebrafish larvae exposed to 125μM VPA during the embryonic period and treated with different RISP concentrations. Triplicate data (n=21-24 larvae/group) expressed in millimeters (mm) as median and interquartile ranges. *p<0.05 compared to the vehicle group. (E)- Social interaction test for socialization profile analysis of 7 dpf zebrafish larvae exposed to 125μM VPA during the embryonic period and treated with different RISP concentrations. Triplicate data (n=18-22 larvae/group) expressed in seconds (s) as median and interquartile ranges.

**Prolonged RISP treatment, but not with CBD, induced changes in the swimming bladder development of zebrafish larvae exposed or not to VPA during the embryonic period.**

Figure Sup2 (A-C) presents the results of the swimming bladder morphological evaluation in 7 dpf zebrafish larvae exposed to 125μM VPA during the embryonic period and treated for a prolonged period (2 to 7 dpf), with previously standardized 1μM of RISP or 0.06μM of CBD concentrations. The analysis of the swimming bladder area values using the Two-way ANOVA test did not demonstrate a statistical effect for VPA exposure (F_1,34_=3.53; p>0.05). But it did show a statistical effect for the treatment (F_2, 34_=37.39; p<0.001). Also, the analysis returned no significant statistical interaction between the exposure × treatment variables (F_2,34_=0.003; p>0.05). Thus, it is possible to observe in Figure Sup2B a statistically significant increase in the swimming bladder area of animals exposed or not to 125μM VPA during the embryonic period, and treated with 1 μM of RISP from 2 to 7 dpf compared to the other groups (p<0.001). Figure Sup2C shows representative images of the prolonged treatment effect with 1 μM RISP or 0.06μM CBD concentrations on the swimming bladder development.


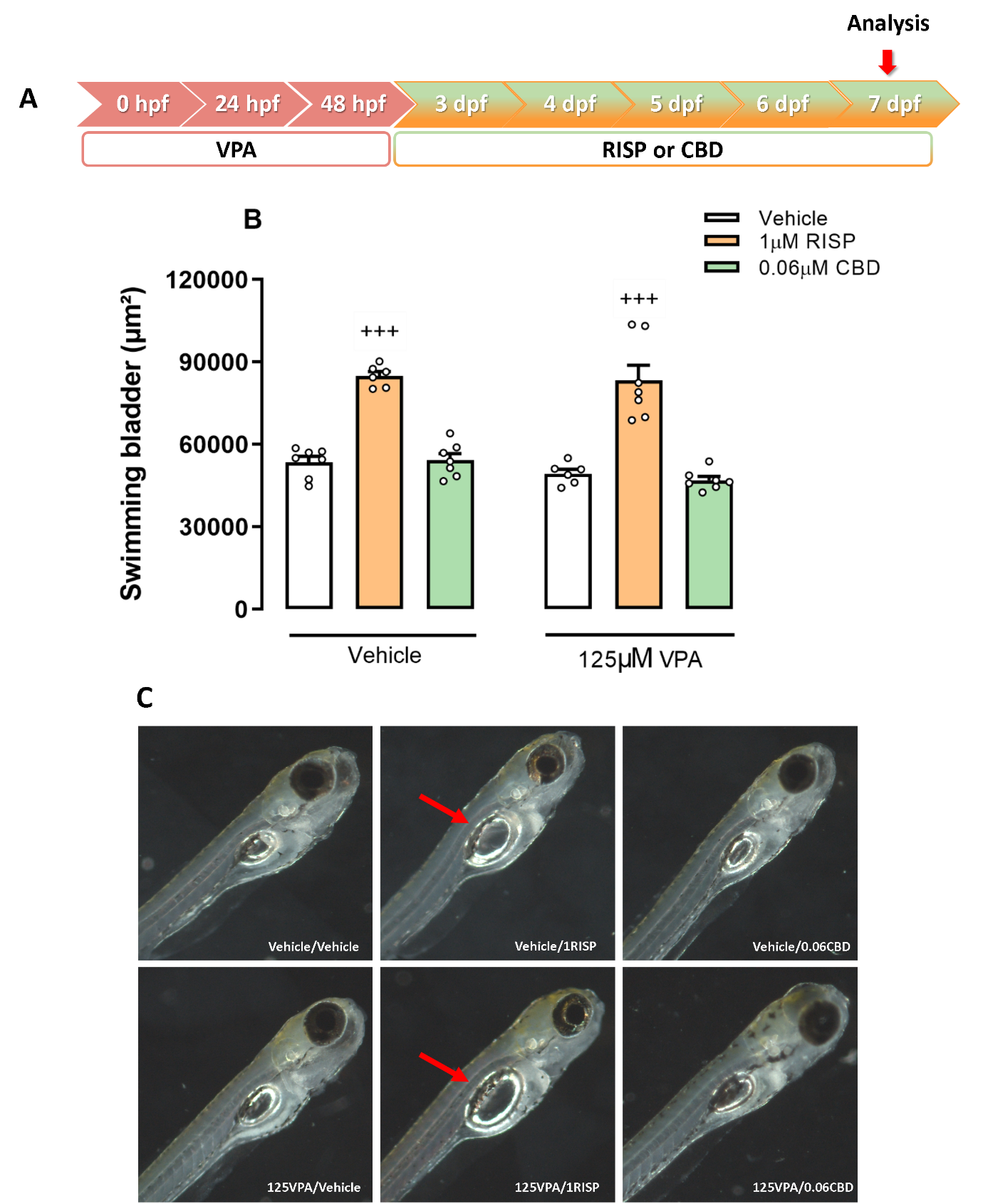


**Supplementary Figure 2:** Morphological analysis of the larval swimming bladder. (A) Schematic representation of the experimental design. (B) Assessment of swim bladder area (μm²)of zebrafish larvae (7 dpf) exposed to VPA during the embryonic period and treated for a prolonged time (from 2 to 7 dpf) with standardized RISP and CBD concentrations. (C) Representative images of the prolonged treatment effect on swimming bladder development (red arrow: swimming bladder with an increase in the inflated area). Data from one replicate (n=6-7 larvae/group) and expressed as mean ± SEM. Two-way ANOVA followed by Duncan's post hoc test. ^+++^p<0.001 compared to the Vehicle/Vehicle; Vehicle/0.06CBD; 125VPA/Vehicle and 125VPA/0.06CBD groups.
